# Supplementary material for: Increasing soil nitrous acid emissions driven by climate and fertilization change aggravate global ozone pollution
Source: Nat Commun. 2025 Mar 12;16:2463. doi: 10.1038/s41467-025-57161-6 (PMC11904233; doi:10.1038/s41467-025-57161-6)
Supplement: Supplementary file 1 — Supplementary Information [file 41467_2025_57161_MOESM1_ESM.pdf]

Supplementary Information for

Increasing soil nitrous acid emissions driven by climate and fertilization change aggravate global ozone pollution

Yanan Wang[1,#], Qinyi Li[1,2,#], Yurun Wang[1], Chuanhua Ren[1,3], Alfonso Saiz-Lopez[4], Likun Xue[2], Tao Wang[1,*]

1 Department of Civil and Environmental Engineering, The Hong Kong Polytechnic University, Hong Kong, China

2 Environment Research Institute, Shandong University, 266237 Qingdao, China

3 Joint International Research Laboratory of Atmospheric and Earth System Sciences, School of Atmospheric Sciences, Nanjing University, 210023 Nanjing, China

4 Department of Atmospheric Chemistry and Climate, Institute of Physical Chemistry Blas Cabrera, Spanish National Research Council (CSIC), 28006 Madrid, Spain

[#] These authors contributed equally.

* Correspondence to Tao Wang (tao.wang@polyu.edu.hk).

This PDF file includes 33 pages, 1 supplementary text1, 19 figures, and 8 tables.

The list of Supplementary Texts S1, Figure S1 to S19, and Table S1 to S8:

Supplementary Texts

S1. Uncertainty analysis

Our study estimates the long-term trends of global soil HONO emissions based on comprehensive dataset of existing global soil HONO emission measurements and quantifies their subsequent impact on global air quality. However, we acknowledge potential uncertainties in our estimates HONO emissions, particularly in regions lacking direct measurements of the soil HONO flux such as in North America. Only when such measurements become available can we compare our estimated emissions with observed values to quantify the uncertainties. Additionally, our estimates use the average emissions from soil samples of different land-use types within specific

latitudinal bands and longitudinal columns to represent corresponding regional and land-use type emissions. This approach introduces uncertainty due to heterogeneous emissions across different locations. Moreover, our parameterization does not include factors such as soil pH and texture, adding to the uncertainty in our estimates of HONO emissions. Furthermore, when handling soil emissions post-fertilization soil emissions, we directly used MERRA2 soil moisture data, which may not fully reflect changes in soil moisture due to irrigation, thereby introducing an uncertainty in our estimation of global soil HONO emissions. Despite these limitations, our soil HONO emission estimates consider all controlling factors on which there is consensus, including soil temperature, soil water content, land cover, fertilizer consumption, and soil pH (only for fertilizer-induced soil HONO emission). Therefore, we believe that our estimated global soil HONO emissions have included most (if not all) available information across various regions and represent the state-of-the-art knowledge on soil HONO sources.

In addition to the uncertainties in soil HONO emissions, the production and emission of HONO involve multiple processes and environmental factors, including traffic emissions, biomass burning, and atmospheric chemical reactions. For these sources, the formulas and parameter values used in the estimation process also carry uncertainties. To evaluate the influence of parameter selection for these HONO sources on the impact of soil HONO emissions, we conducted sensitivity tests by using the maximum and minimum values for the parameters (Table S7) of HONO sources. The results (Table S8) indicate that if we select smaller parameters, the impact of soil HONO emissions on air quality would be more pronounced. To ensure a conservative estimate of the impact of soil HONO emissions, the parameters used in our study, as shown in Table S7, are at their maximum or median values, and we have not used the minimum values to evaluate the impact of soil HONO emissions.

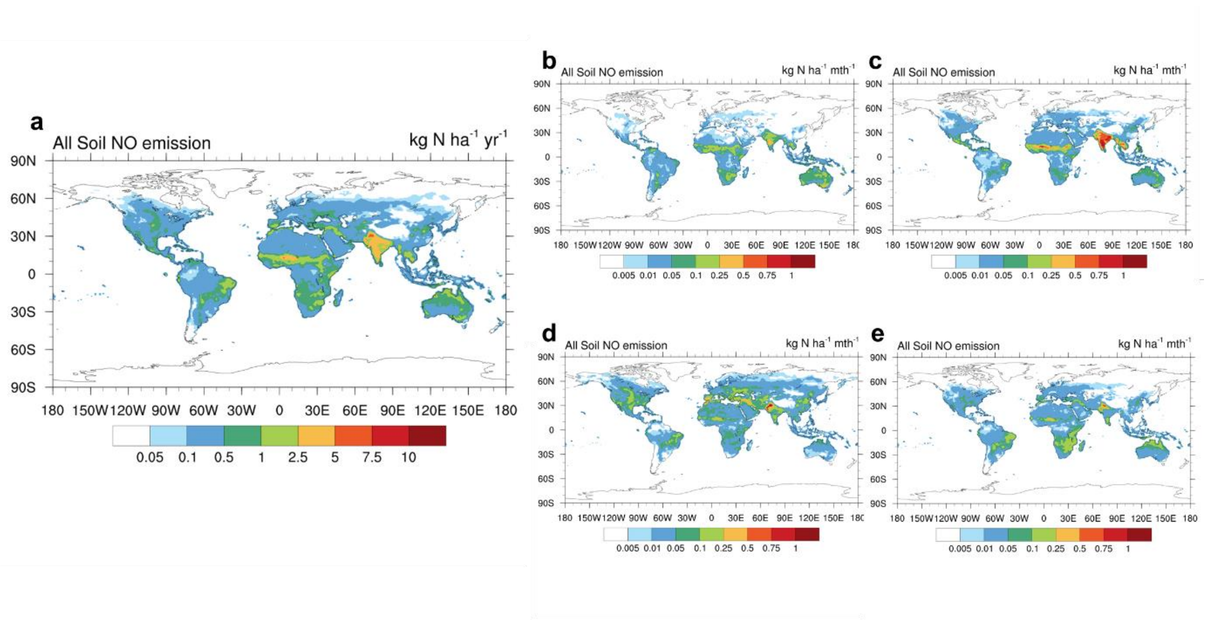

Fig. S1 Global soil NO emission. (a) Soil NO emission in 2016. (b-e) Monthly variation of soil NO emissions.

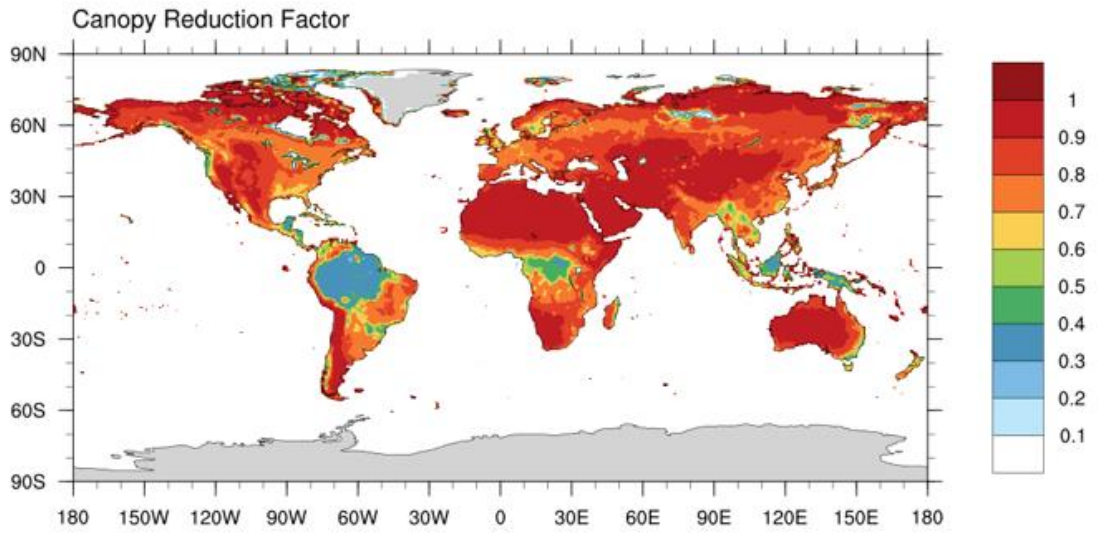

Fig. S2 Annual average of the canopy reduction factor (CRF) in 2016. A larger CRF indicates a smaller canopy uptake.

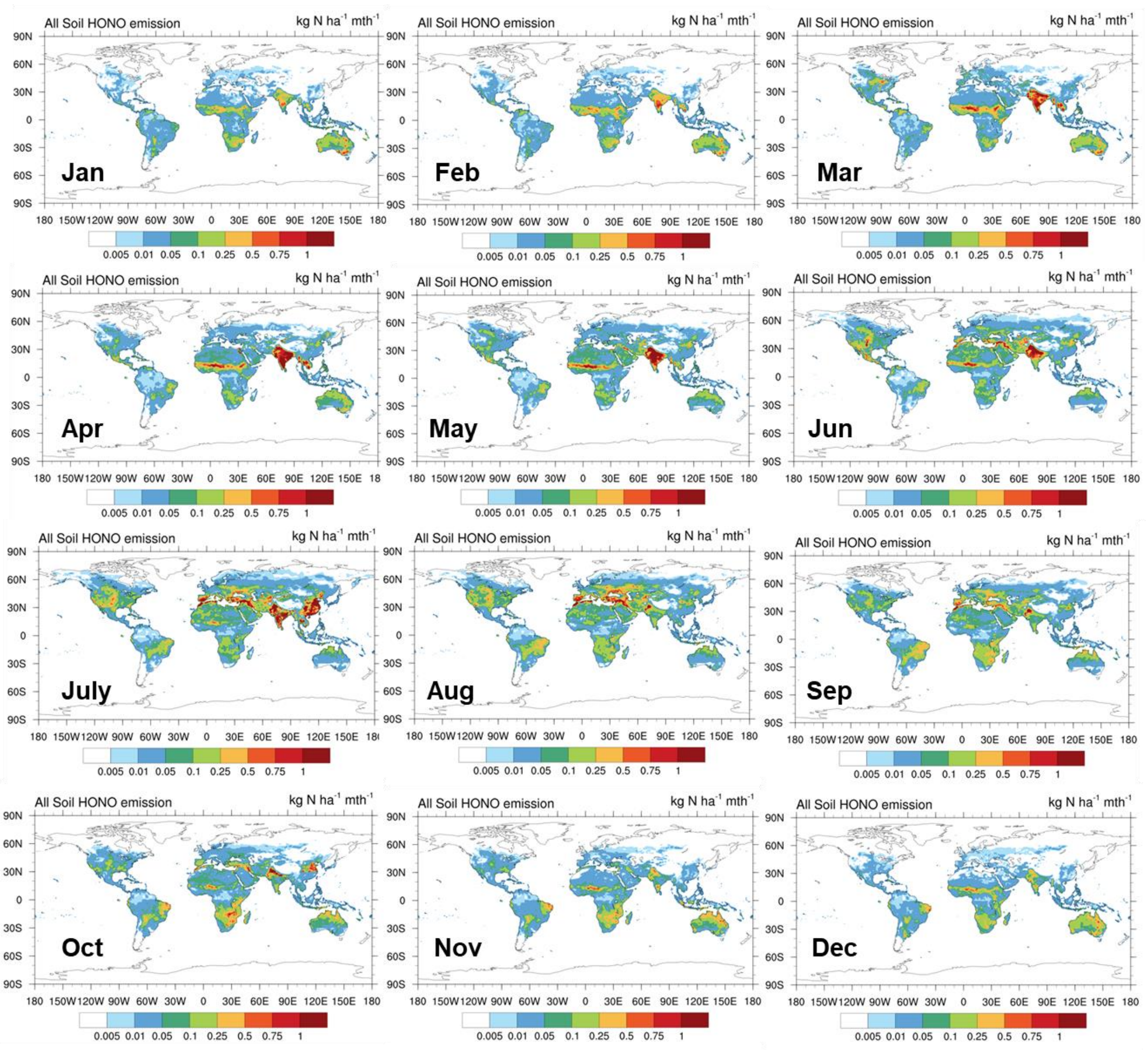

Fig. S3 Monthly variation of soil HONO emissions in 2016.

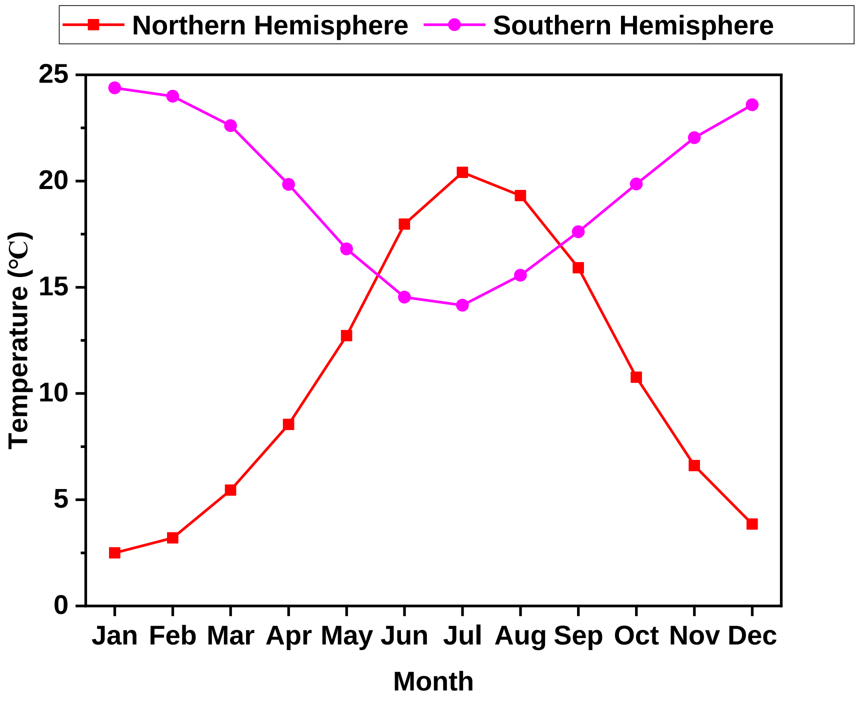

Fig. S4 The monthly average variations of soil temperature in 2016 (Data from MERRA2).

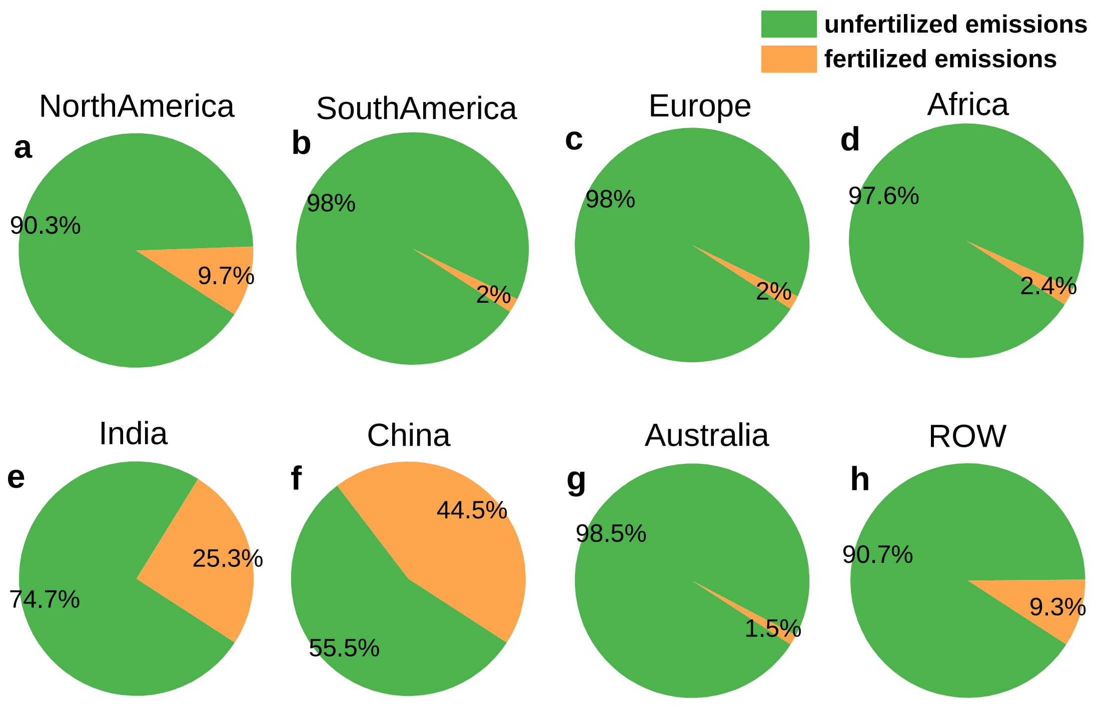

Fig. S5 The proportion of HONO emissions from fertilized and unfertilized soils in different counties or regions. (a–h) The percentage of HONO emissions originating

from fertilized and unfertilized soils in North America, South America, Europe, Africa, India, China, Australia, and the rest of the world (ROW), respectively.

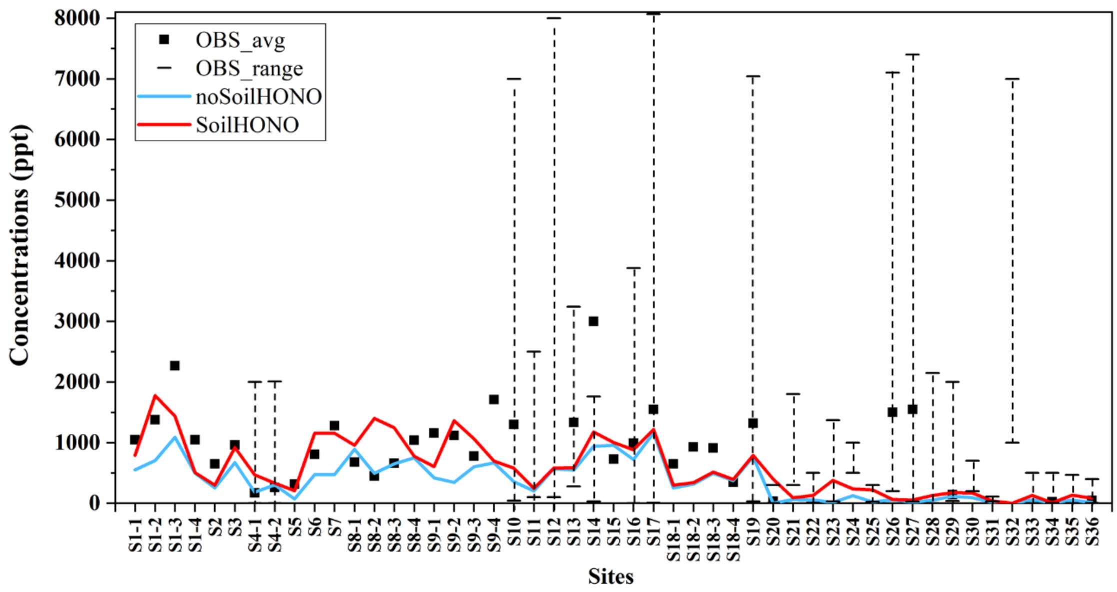

Fig. S6 Comparison of simulated and observed HONO concentrations at 36 observation sites. The blue and red lines illustrate the simulated average values during the campaign period for the noSoilHONO and SoilHONO cases, respectively. The black squares and whiskers represent the observed average mixing ratios and the range, respectively. The information of these sites is shown in Table S2.

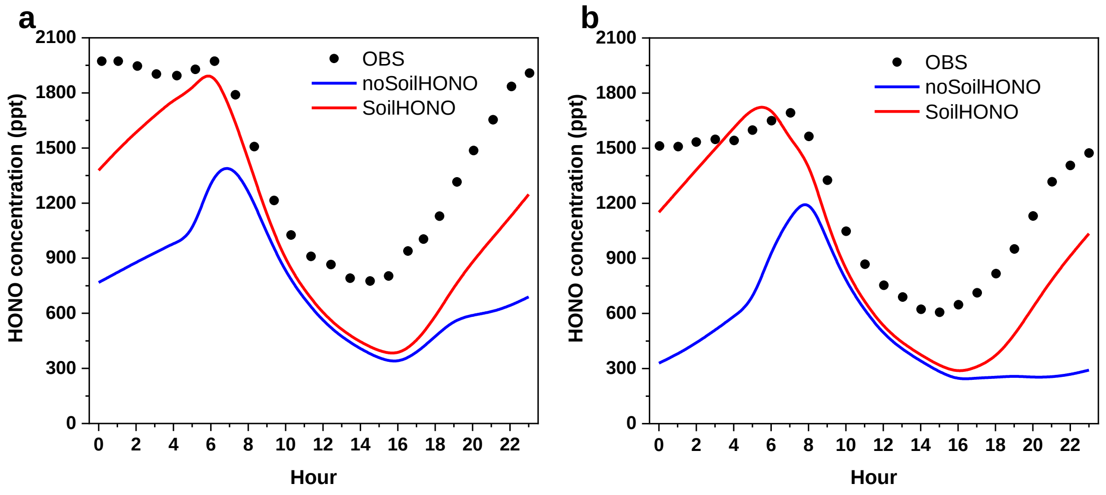

Fig S7. Observed and simulated diurnal variations of HONO mixing ratios at (a) Beijing and (b) Jinan sites in 2016. The black dots represent observed mixing ratios, and the blue and red lines represent the simulated results of noSoilHONO and SoilHONO cases, respectively. The observation at Beijing is reported by Wang et al. [1]; that at Jinan by Li et al. [2].

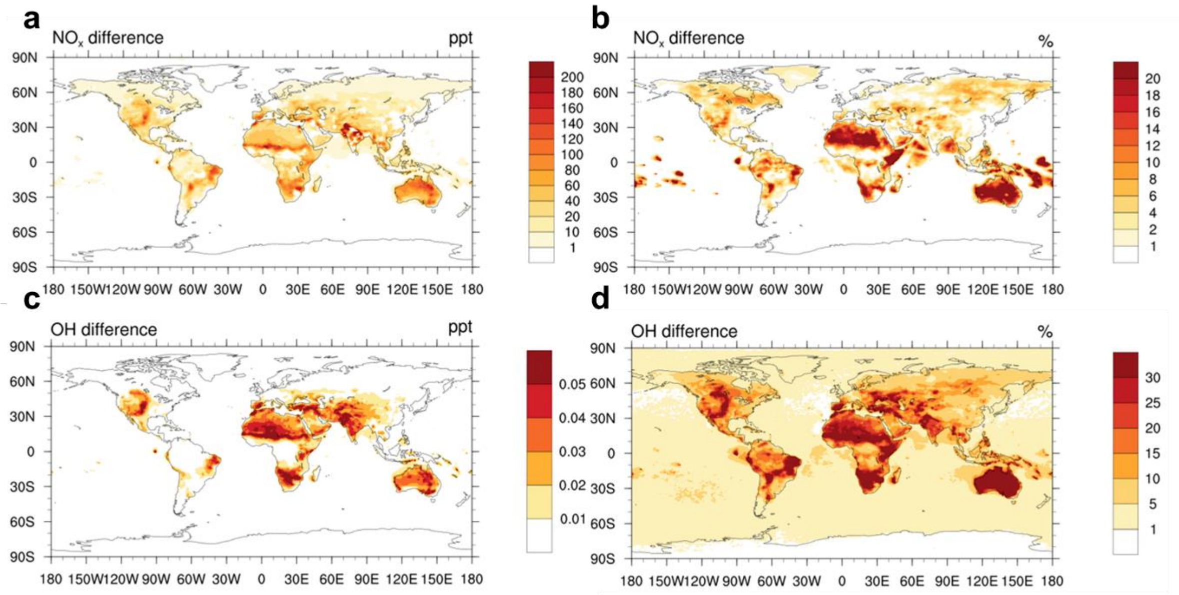

Fig. S8 Global impact of soil HONO emissions on NO_x and OH in 2016. (a) and (b) represent the absolute and relative increases of OH due to soil HONO emissions,

respectively. (c) and (d) represent the absolute relative increases of NO_x due to soil HONO emissions, respectively.

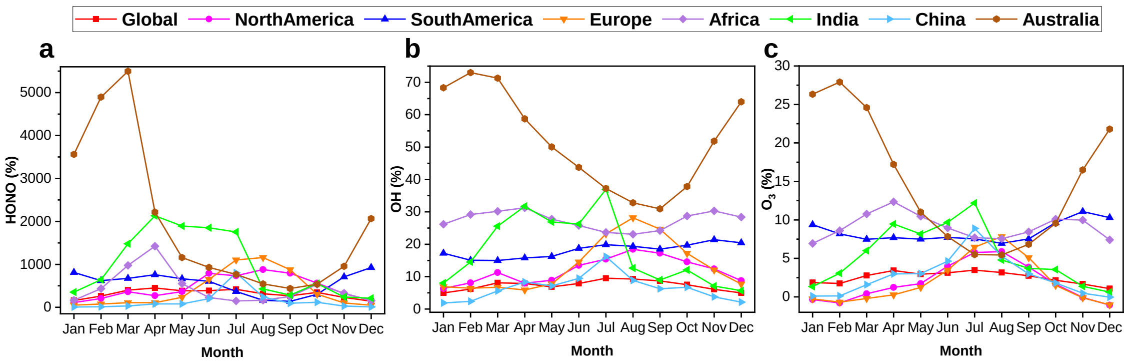

Fig. S9 Soil HONO emissions induced monthly average relative increase of the atmospheric concentrations of (a) HONO, (b) OH, and (c) O_3 in 2016.

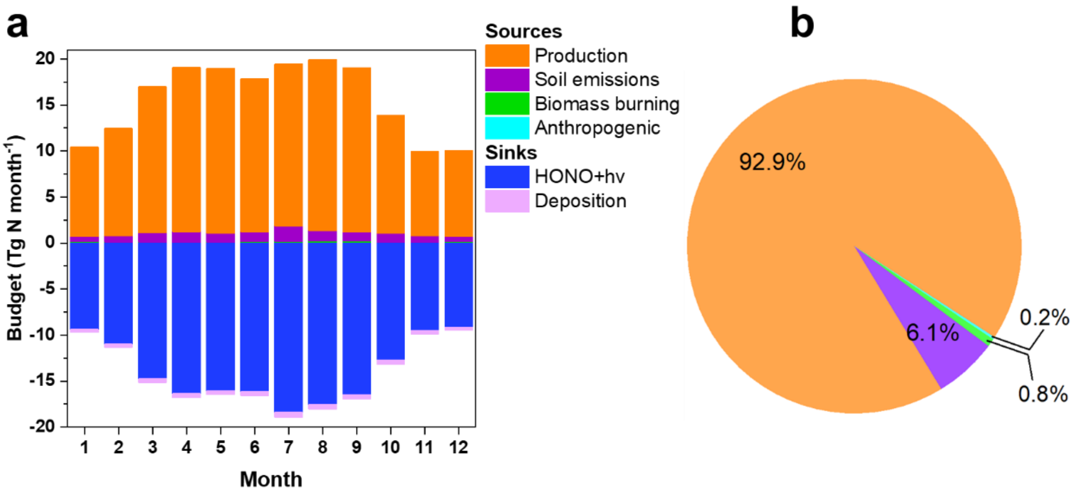

Fig. 10 Tropospheric HONO budget and relative contribution from different sources in 2016. (a) The contributions of different sources to tropospheric HONO in different months of 2016. (b) Annual averaged relative contribution of each HONO source to tropospheric HONO.

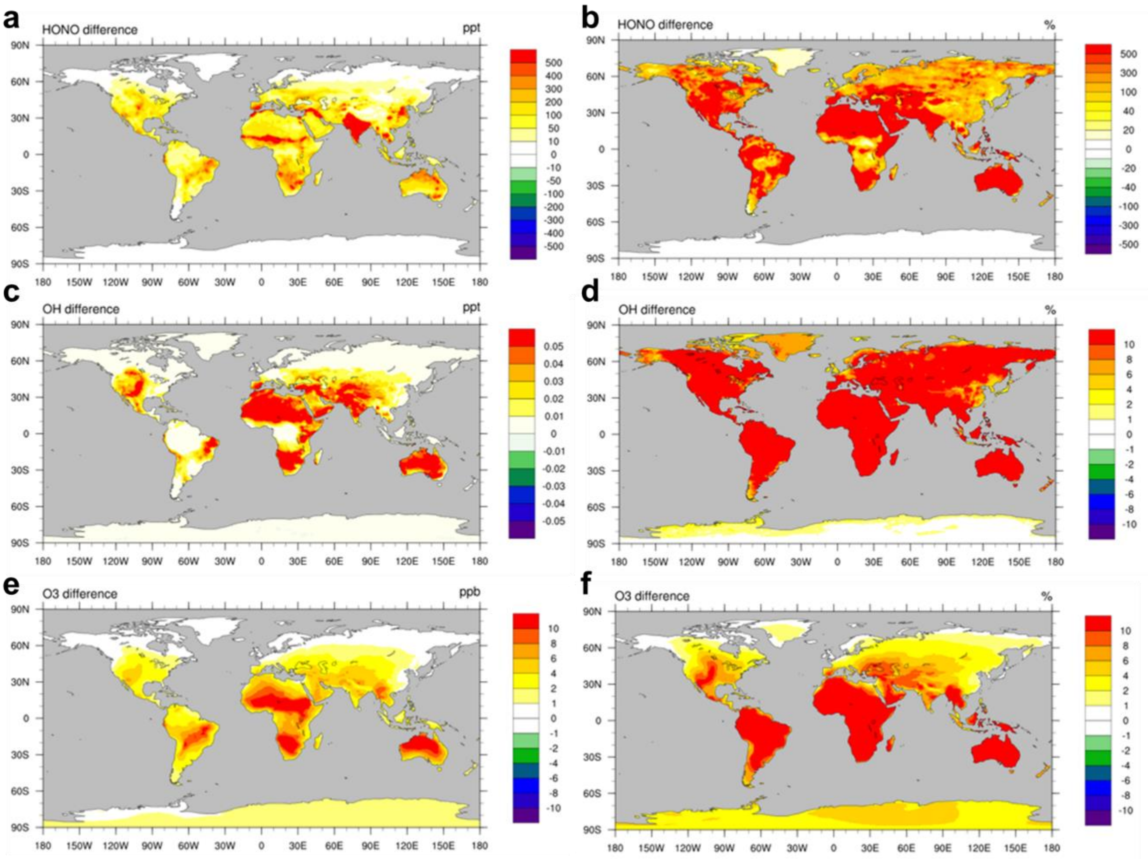

Fig. S11 Soil HONO and NO emissions impact on air quality. (a, c, and e) represent the absolute contributions of soil emissions to HONO (ppt), OH (ppt), and O_3 (ppb) concentrations, respectively. (b, d, and f) represent the relative contributions of soil emissions to these species.

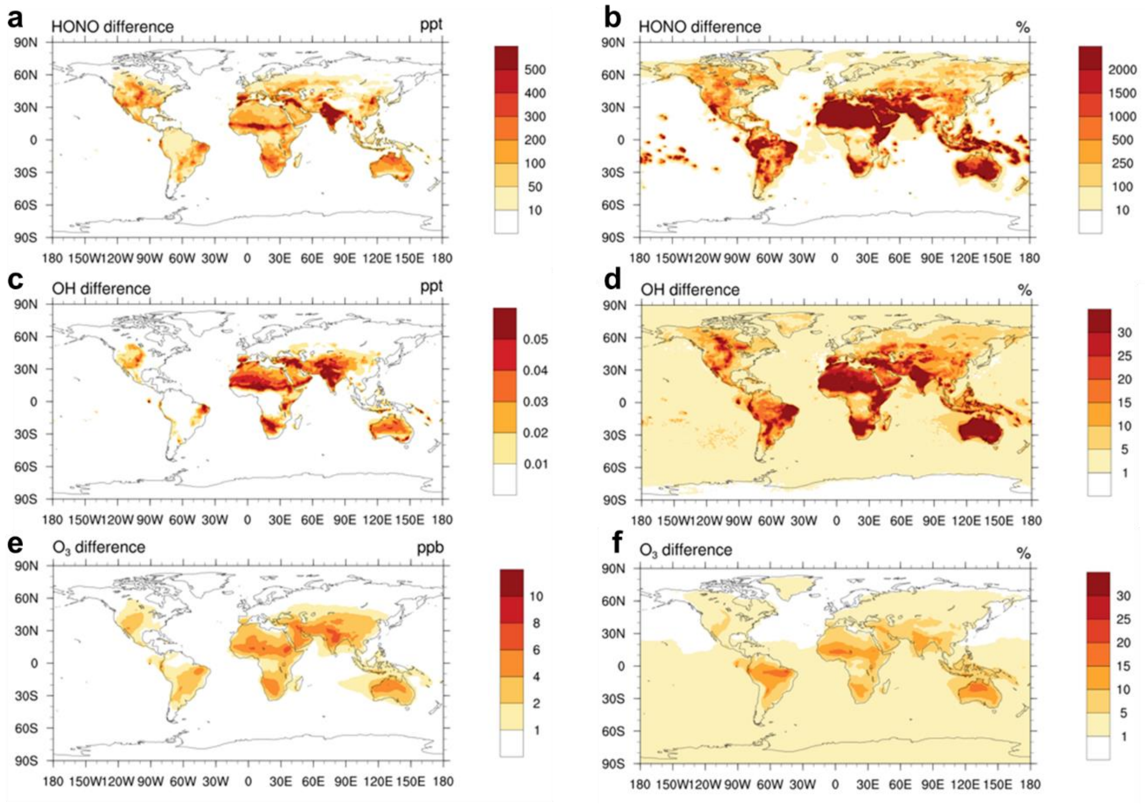

Fig. S12 Impact of soil HONO emission on air quality in 1981. (a, c, e) Absolute increases in HONO (pptv) OH (pptv), and O_3 (ppbv) concentrations owing to soil HONO emissions, respectively. (b, d, f) Relative increases in these species owing to soil HONO emissions.

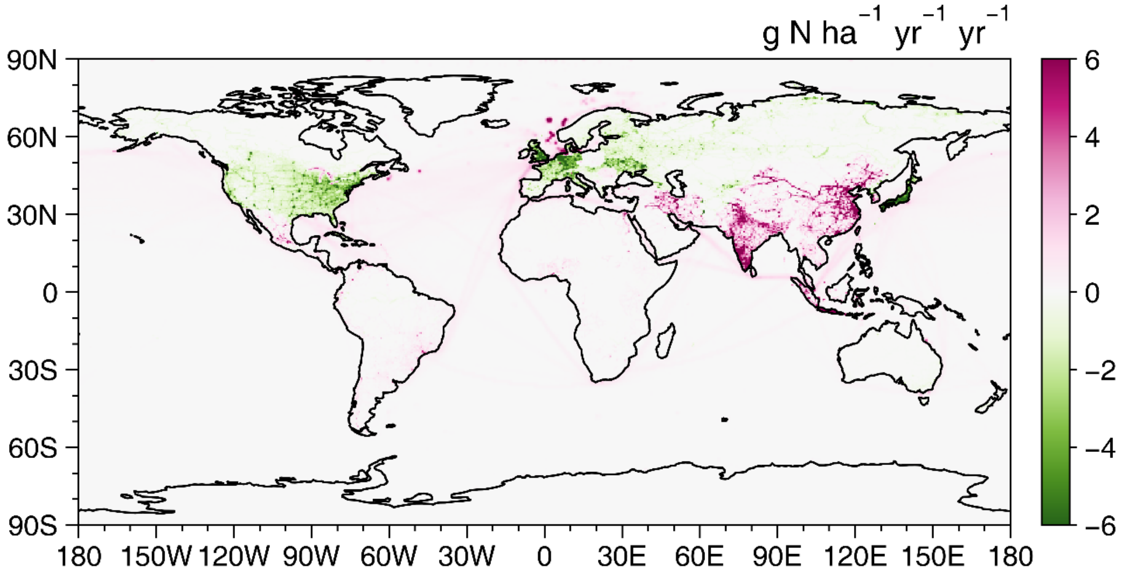

Fig. S13 Change rate of anthropogenic direct vehicle HONO emissions during 1980 to 2016. The figures display the results of linear regression analysis conducted on the emissions data for each grid from 1980 to 2015. The slopes of the regression are visually presented in the figures.

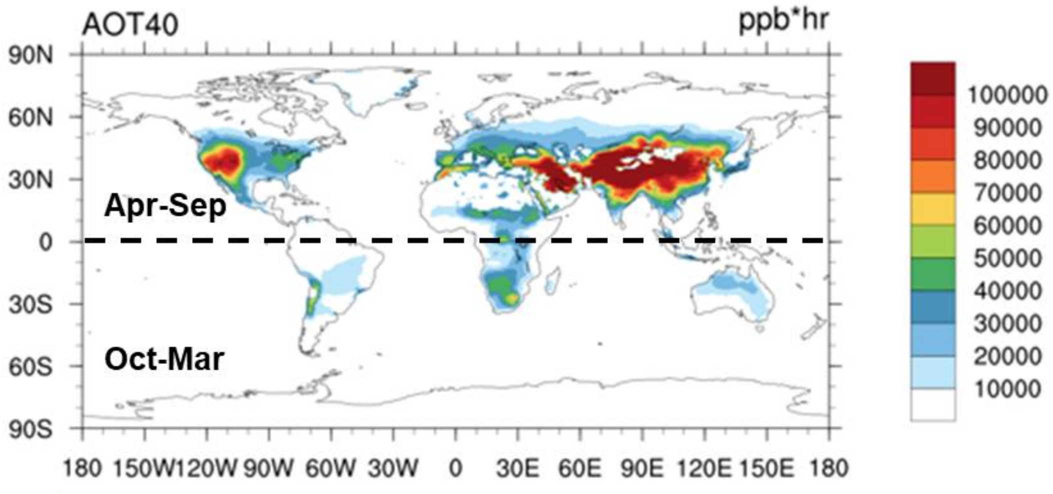

Fig. S14 Global distribution of AOT40 (accumulated O_3 concentration over a threshold of 40 ppbv in the daytime) in 2016.

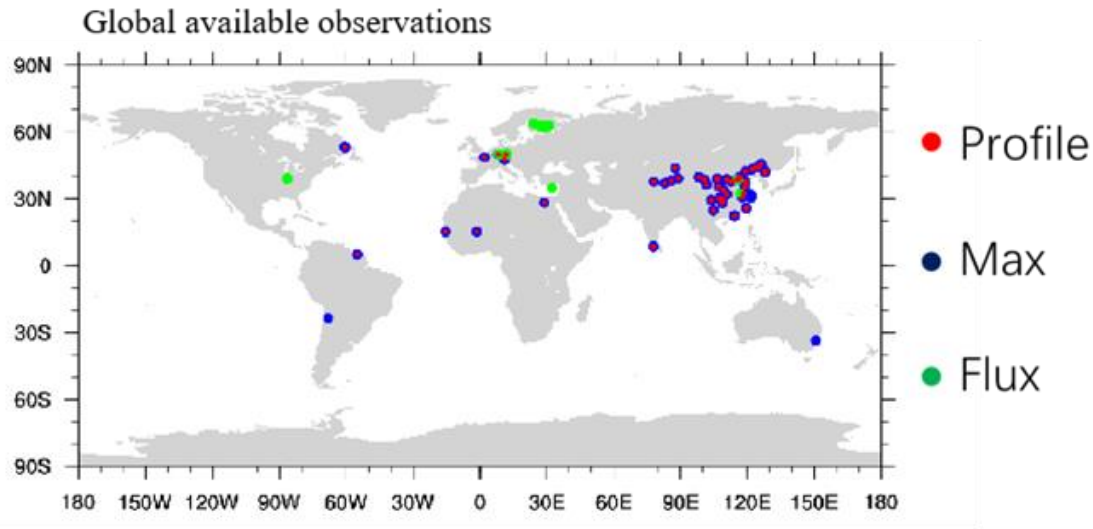

Fig. S15 Locations of soil samples for HONO emissions in literature.

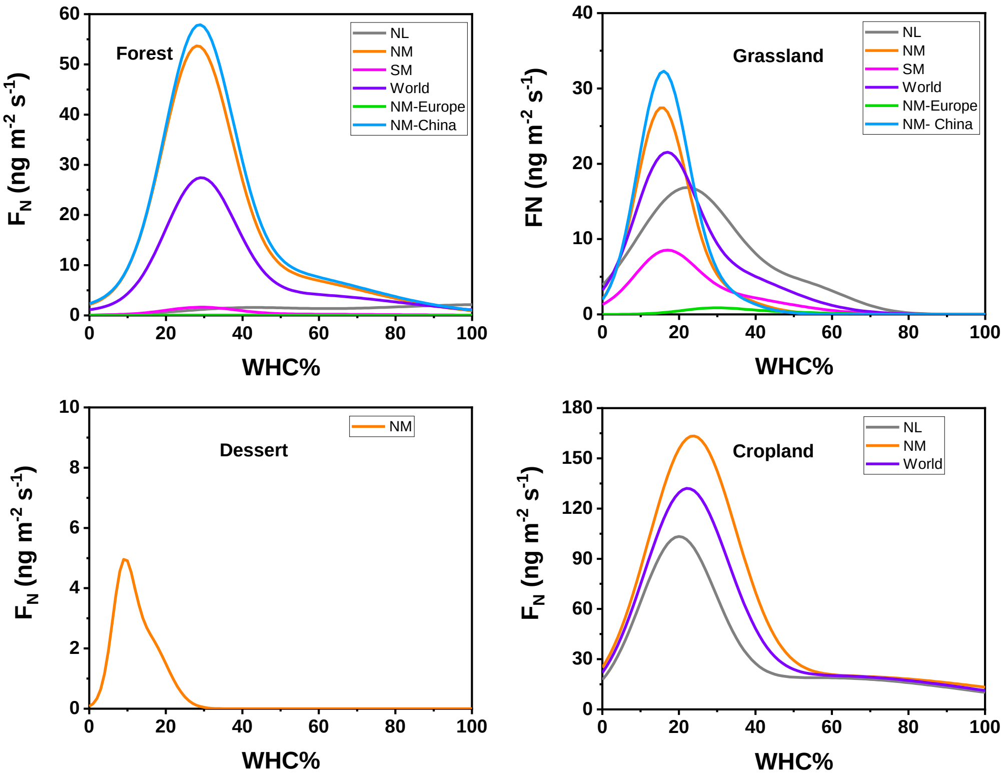

Fig. S16 The soil water content (SWC) dependence of fitted soil HONO emissions in different regions. NL, NM, and SM represent north low, north middle, and south middle latitude, respectively.

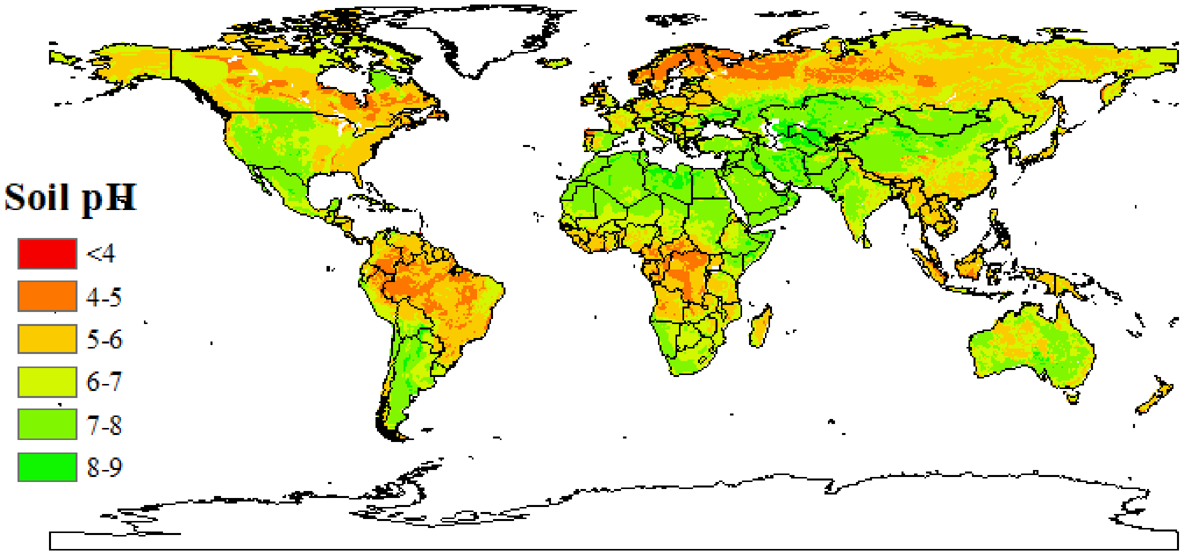

Fig. S17 Global soil pH (IGBP-DIS Soil Data)[3].

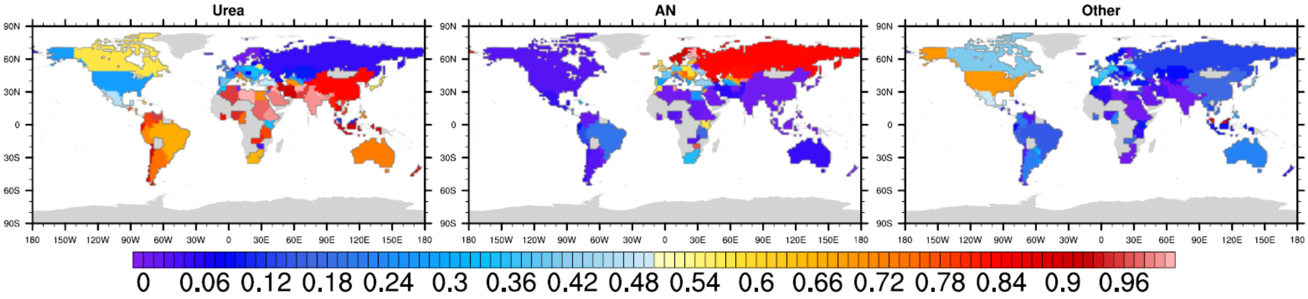

Fig. S18 The proportion of different fertilizer types in each country in 2015. AN and other represent the proportions of ammonium nitrate and fertilizers other than urea and ammonium nitrate.

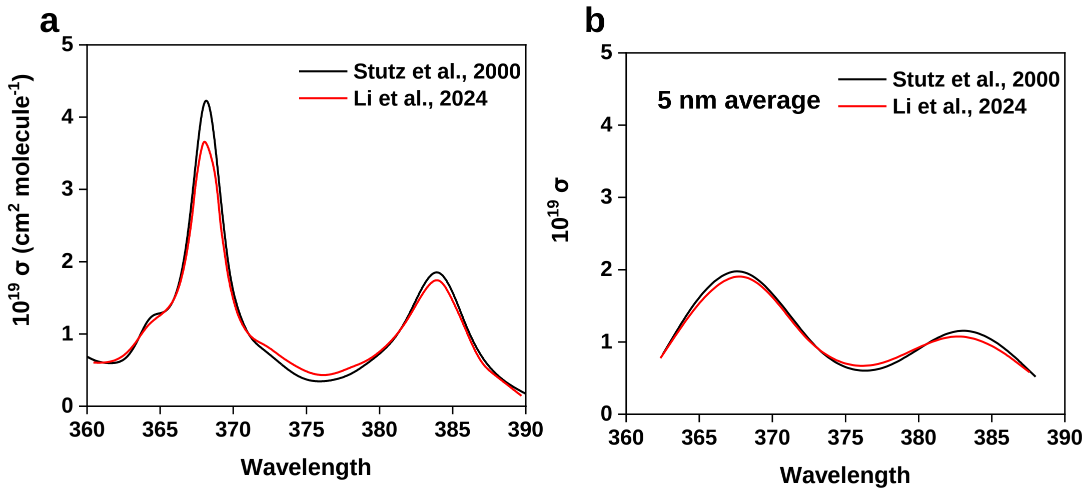

Fig. S19 Comparison of HONO absorption cross section (360–390 nm) from Stutz et al. [4] (used in CAM-Chem model) and Li et al.[5]. (a) shows the original HONO absorption cross section values, while (b) presents the averaged values with a 5 nm interval (used in CAM-Chem).

Table S1 The specific timing for fertilization in different regions.

Regions	Starting day	
	Crop intensity = 1	Crop intensity = 2
AMNM	03/20	03/10_08/15
AMNL	06/10	05/20_10/20
AMSL	01/10	03/01_10/01
AMSM	08/15	02/20_08/15
EANM	03/01	03/10_09/20
EANL	03/30	03/10_08/20
EASL	10/01	02/20_09/10

EASM	09/25	
ASNM	04/10	07/01_11/10
ASNL	06/20	03/10_10/10
ASSL	11/20	11/20_04/10
ASSM	08/01	02/20_08/20

Table S2 The 36 globally distributed HONO observation sites used for model validation.

Sites	Longitude	Latitude	Reference
S1	116.34°E	40.01°N	Wang et al., 2017[1]
S2	113.03°E	23.5°N	Li et al., 2012[6]
S3	103.88°E	30.40°N	Yang et al., 2021[7]
S4	118.98°E	37.76°N	Gu et al., 2020[8]
S5	120.69°E	36.37°N	Yang et al., 2021[9]
S6	106.50°E	29.62°N	Sun et al., 2020[10]
S7	106.59°E	29.43°N	Sun et al., 2020[10]
S8	118.95°E	32.12°N	Liu et al., 2019[11]
S9	117.05°E	36.67°N	Li et al., 2018[2]
S10	121.5°E	31.3°N	Bernard et al., 2016[12]
S11	112.53°E	22.32°N	Cheng et al., 2013[13]

S12	112.92°E	22.71°N	Fu et al., 2019[14]
S13	116.32°E	39.99°N	Hou et al., 2016[15]
S14	115.73°E	39.15°N	Xu et al., 2019[16]
S15	116.3°E	39.99°N	Ma et al., 2019[17]
S16	121.48°E	31.3°N	Nan et al., 2017[18]
S17	119.9°E	31.8°N	Shi et al., 2020[19]
S18	113.93°E	22.3°N	Xu et al., 2015[20]
S19	118.71°E	32.21°N	Zheng et al., 2020[21]
S20	32.38°E	34.96°N	Meusel et al., 2016[22]
S21	0.21°W	51.52°N	Lee et al., 2016[23]
S22	2.21°E	48.72°N	Michoud et al., 2014[24]
S23	118.97°W	35.35°N	VandenBoer et al., 2014[25]
S24	127.32°E	37.31°N	Kim et al., 2015[26]
S25	120.63°W	38.98°N	Ren et al., 2011[27]
S26	139.8°E	35.7°N	Nakashima et al., 2017[28]
S27	85.36°E	27.72°N	Yu et al., 2009[29]
S28	95.35°W	29.69°N	Wong et al., 2011[30]
S29	6.39°E	50.91°N	Kleffmann et al., 2005[31]
S30	8.43°E	49.09°N	Kleffmann et al., 2003[32]
S31	11°E	48°N	Acker et al., 2006[33]

S32	70.67°E	33.45°S	Elshorbany et al., 2009[34]
S33	2.207°E	48.718°N	Michoud et al., 2012[35]
S34	156.65°W	71.32°N	Vilena et al., 2011[36]
S35	82.917°W	42.034°N	Wentzell et al., 2010[37]
S36	73.9°W	44.4°N	Zhou et al., 2007[38]

Table S3 The concentrations of HONO, OH, and O_3 in different cases in 1981 and 2016.

Year	Case	emissions	HONO (pptv)	OH (pptv)	O_3 (ppbv)
1981	noSoilHONO	-	7.68	0.056	28.79
	SoilHONO	8.82	32.63	0.061	29.69
2016	noSoilHONO	-	9.19	0.063	30.45
	SoilHONO	11.52	36.99	0.068	31.22

Table S4 Global soil HONO emissions measurements, including both laboratory and field studies. Among them, only data reporting the SWC dependence of soil HONO and NO emissions are used for subsequent parameterization (110 laboratory measurements, Table S5)

No.	Country	Latitude	Longitude	Land use	Reference
1	Australia	-33.61	150.63	Broadleaf forest	Oswald et al., 2013[39]
2	Suriname	5.08	-55	Tropical rain forest	Oswald et al., 2013[39]
3	Germany	47.8	11.01	Coniferous forest	Oswald et al., 2013[39]
4	Germany	50.09	11.52	Coniferous forest	Oswald et al., 2013[39]
5	Australia	-33.57	150.77	Pasture	Oswald et al., 2013[39]
6	Senegal	15.4	-15.43	Open woody	Oswald et al., 2013[39]
7	Mali	15.34	-1.48	Open woody	Oswald et al., 2013[39]
8	Germany	49.97	8.16	Grassland	Oswald et al., 2013[39]
9	Germany	47.79	11	Pasture	Oswald et al., 2013[39]
10	Chile	-23.48	-68.03	Desert	Oswald et al., 2013[39]
11	France	48.85	1.97	Cropland	Oswald et al., 2013[39]
12	Germany	49.97	8.16	Cropland	Oswald et al., 2013[39]
13	China	38.09	85.55	Cropland	Oswald et al., 2013[39]
14	China	38.1	85.55	Cropland	Oswald et al., 2013[39]
15	China	37.05	82.71	Cropland	Oswald et al., 2013[39]
16	China	37.69	77.89	Desert	Oswald et al., 2013[39]
17	China	39.27	88.91	Cropland	Oswald et al., 2013[39]
18	Germany	49.97	8.16	Cropland	Wu et al., 2019[40]
19	India	8.7	77.87	Cropland	Wu et al., 2019[40]
20	India	8.7	77.45	Cropland	Wu et al., 2019[40]
21	Egypt	28.35	28.86	Cropland	Wu et al., 2019[40]
22	China	32.12	110.7	Cropland	Wu et al., 2019[40]
23	China	37.76	118.99	Grassland	Wu et al., 2019[40]
24	Ireland	53.31	-60.54	Cropland	Wu et al., 2019[40]
25	Ireland	53.31	-60.54	Cropland	Wu et al., 2019[40]
26	Ireland	53.31	-60.54	Grassland	Wu et al., 2019[40]
27	China	39.8	116.47	Cropland	Wu et al., 2019[40]
28	China	39.8	116.47	Cropland	Wu et al., 2019[40]
29	China	30.75	121.28	Cropland	Wu et al., 2022[41]
30	China	30.91	121.09	Cropland	Wu et al., 2022[41]
31	China	31.08	121.84	Cropland	Wu et al., 2022[41]
32	China	31.23	121.18	Cropland	Wu et al., 2022[41]

33	China	31.32	121.83	Cropland	Wu et al., 2022[41]
34	China	31.45	121.59	Cropland	Wu et al., 2022[41]
35	China	31.57	121.83	Cropland	Wu et al., 2022[41]
36	China	31.8	121.35	Cropland	Wu et al., 2022[41]
37	China	31.05	120.89	Cropland	Wu et al., 2022[41]
38	China	30.92	121.42	Cropland	Wu et al., 2022[41]
39	China	31.48	121.36	Forest	Wu et al., 2022[41]
40	China	30.83	121.28	Forest	Wu et al., 2022[41]
41	China	30.9	121.56	Forest	Wu et al., 2022[41]
42	China	31.07	121.18	Forest	Wu et al., 2022[41]
43	China	31.17	121.65	Forest	Wu et al., 2022[41]
44	China	31.32	121.55	Forest	Wu et al., 2022[41]
45	China	31.4	121.27	Forest	Wu et al., 2022[41]
46	China	31.07	120.99	Forest	Wu et al., 2022[41]
47	China	31.65	121.64	Forest	Wu et al., 2022[41]
48	China	31.72	121.36	Forest	Wu et al., 2022[41]
49	China	31.24	121.65	Forest	Wu et al., 2022[41]
50	China	31.15	121.08	Grassland	Wu et al., 2022[41]
51	China	30.99	121.27	Grassland	Wu et al., 2022[41]
52	China	31.23	121.27	Grassland	Wu et al., 2022[41]
53	China	31.41	121.66	Grassland	Wu et al., 2022[41]
54	China	30.99	121.08	Grassland	Wu et al., 2022[41]
55	China	31	121.75	Grassland	Wu et al., 2022[41]
56	China	31.15	121.45	Grassland	Wu et al., 2022[41]
57	China	31.31	121.27	Grassland	Wu et al., 2022[41]
58	China	31.4	121.45	Grassland	Wu et al., 2022[41]
59	China	31.72	121.55	Grassland	Wu et al., 2022[41]
60	China	31.56	121.55	Shrubland	Wu et al., 2022[41]
61	China	30.92	121.93	Shrubland	Wu et al., 2022[41]
62	China	31.24	121.46	Bare land	Wu et al., 2022[41]

63	China	31.08	121.46	Bare land	Wu et al., 2022[41]
64	Finland	63.92	24.05	Pristine peatlands	Maljanen et al., 2013[42]
65	Finland	62.78	30.93	Pristine peatlands	Maljanen et al., 2013[42]
66	Finland	62.78	30.88	Pristine peatlands	Maljanen et al., 2013[42]
67	Finland	63.9	23.97	Drained peatlands	Maljanen et al., 2013[42]
68	Finland	63.9	23.93	Drained peatlands	Maljanen et al., 2013[42]
69	Finland	63.85	23.85	Drained peatlands	Maljanen et al., 2013[42]
70	Finland	63.9	23.97	Drained peatlands	Maljanen et al., 2013[42]
71	Finland	63.9	23.93	Pine forest	Maljanen et al., 2013[42]
72	Finland	62.65	27.05	Pine forest	Maljanen et al., 2013[42]
73	Finland	62.78	30.93	Pine forest	Maljanen et al., 2013[42]
74	Finland	62.43	28.63	Spruce forest	Maljanen et al., 2013[42]
75	Cyprus	34.9638	32.3778	Bare soil	Meusel et al., 2018[43]
76	America	39.08	-86.47	Forest	Mushinski et al., 2019[44]
77	America	39.08	-86.47	Forest	Mushinski et al., 2019[44]
78	Germany	49.99	8.22	Cropland	Bao et al., 2022[45]
79	America	39.17	-86.47	Cropland	Scharko et al., 2015[46]
80	America	39.16	-86.53	Grassland	Scharko et al., 2015[46]
81	America	39.16	-86.53	Grassland	Scharko et al., 2015[46]
82	Finland	63.15	27.3	Cropland	Bhattarai et al., 2018[47]
83	Finland	63.15	27.3	Cropland	Bhattarai et al., 2018[47]
84	Finland	63.15	27.3	Cropland	Bhattarai et al., 2018[47]
85	Germany	50.14	11.87	Forest	Sörgel et al., 2015[48]
86	Germany	50.14	11.87	Forest	Sörgel et al., 2015[48]
87	Germany	50.14	11.87	Shrubland	Sörgel et al., 2015[48]
88	Germany	50.14	11.87	Grassland	Sörgel et al., 2015[48]
89	China	32.43	116.79	Cropland	Tang et al., 2020[49]
90	China	38.67	115.25	Cropland	Tang et al., 2019[50]
91	China	38.67	115.25	Cropland	Xue et al., 2019[51]
92	China	43.74	87.50	Cropland	Wang et al., 2023[52]

93	China	39.82	98.22	Cropland	Wang et al., 2023[52]
94	China	38.69	100.72	Cropland	Wang et al., 2023[52]
95	China	35.75	106.94	Cropland	Wang et al., 2023[52]
96	China	38.86	110.5	Cropland	Wang et al., 2023[52]
97	China	34.05	109.17	Cropland	Wang et al., 2023[52]
98	China	39.04	106.4	Cropland	Wang et al., 2023[52]
99	China	36.5	101.63	Cropland	Wang et al., 2023[52]
100	China	37.69	112.56	Cropland	Wang et al., 2023[52]
101	China	38.95	115.91	Cropland	Wang et al., 2023[52]
102	China	42.3	118.91	Cropland	Wang et al., 2023[52]
103	China	44.00	123.13	Cropland	Wang et al., 2023[52]
104	China	44.31	125.03	Cropland	Wang et al., 2023[52]
105	China	45.91	126.00	Cropland	Wang et al., 2023[52]
106	China	30.23	107.27	Cropland	Wang et al., 2023[52]
107	China	29.58	103.7	Cropland	Wang et al., 2023[52]
108	China	28.23	108.58	Cropland	Wang et al., 2023[52]
109	China	24.97	104.6	Cropland	Wang et al., 2023[52]
110	China	29.89	108.6	Cropland	Wang et al., 2023[52]
111	China	25.89	119.41	Cropland	Wang et al., 2023[52]
112	China	30.78	117.47	Cropland	Wang et al., 2023[52]
113	China	35.56	118.83	Cropland	Wang et al., 2023[52]
114	China	38.66	115.25	Cropland	Wang et al., 2023[52]
115	China	22.43	114.11	Cropland	Wang et al., 2023[52]
116	China	43.83	87.5	Forest	Wang et al., 2023[52]
117	China	39.8	98.22	Forest	Wang et al., 2023[52]
118	China	38.69	100.72	Forest	Wang et al., 2023[52]
119	China	38.86	110.5	Forest	Wang et al., 2023[52]
120	China	34.05	109.17	Forest	Wang et al., 2023[52]
121	China	39.04	106.36	Forest	Wang et al., 2023[52]
122	China	36.49	101.57	Forest	Wang et al., 2023[52]

123	China	37.87	112.66	Forest	Wang et al., 2023[52]
124	China	38.95	115.93	Forest	Wang et al., 2023[52]
125	China	43.22	121.83	Forest	Wang et al., 2023[52]
126	China	42.42	128.07	Forest	Wang et al., 2023[52]
127	China	41.90	127.67	Forest	Wang et al., 2023[52]
128	China	30.23	107.27	Forest	Wang et al., 2023[52]
129	China	29.58	103.7	Forest	Wang et al., 2023[52]
130	China	28.22	108.56	Forest	Wang et al., 2023[52]
131	China	24.97	104.6	Forest	Wang et al., 2023[52]
132	China	29.89	108.6	Forest	Wang et al., 2023[52]
133	China	25.88	119.41	Forest	Wang et al., 2023[52]
134	China	32.59	118.3	Forest	Wang et al., 2023[52]
135	China	35.56	118.83	Forest	Wang et al., 2023[52]
136	China	22.24	113.94	Forest	Wang et al., 2023[52]
137	China	22.48	114.18	Forest	Wang et al., 2023[52]
138	China	22.43	114.1	Forest	Wang et al., 2023[52]
139	China	22.39	114.25	Forest	Wang et al., 2023[52]

Table S5 The studies used for parameterization. The form of shape (peak values) is used to represent the references utilized in calculating the shapes and peak values, respectively. World and LBA means word average and latitude band average, respectively.

	Forest & Shrubland	Grassland	Desert	Unfertilized Cropland	Fertilized Cropland
NH	World[39,52] (World[39,41,52])	World[39,40] (World[39-41]):	World[39] (World[39])	World[39,40,52] (World[39-41,52])	China[53] (China[53])
NM	LBA[39,52] (LBA[39,41,52])<br>Europe[39]: (Europe[39])<br>China[52]: (China[41,52])	LBA[39,40] (LBA[39-41])<br>Europe[39]: (Europe[39])<br>China[40]: (China[40,41])	LBA[39] (LBA[39])	LBA[39,40,52] (LBA[39-41,52])	China[53] (China[53])
NL	LBA[39,52] (LBA[39,52])	LBA[39] (LBA[39])	World[39] (World[39])	LBA[40,52] (LBA[40,52])	China[53] (China[53])
SL	World[39,52] (World[39,41,52])	World[39,40] (World[39-41])	World[39] (World[39])	World[39,40,52] (World[39-41,52])	China[53] (China[53])
SM	World[39,52] (LBA[39])	World[39,40] (LBA[39])	World[39] (World[39])	World[39,40,52] (World[39-41,52])	China[53] (China[53])
SH	World[39,52] (World[39,41,52])	World[39,40] (World[39-41])	World[39] (World[39])	World[39,40,52] (World[39-41,52])	China[53] (China[53])

Table S6 Parameterization schemes of soil HONO emissions as multiple Gaussian functions of the soil water content SWC.

Land use	Region	Peak	$F_{N,max}$	SWC_c	w
Forest	NH	Peak1	24.19	28.97	12.79
		Peak2	4.28	51.17	42.24
	NM	Peak1	47.25	28.01	12.57
		Peak1-Europe	0.069	36.25	49.10
		Peak1-China	50.71	28.52	12.58
		Peak2	8.00	45.34	37.34
		Peak2-Europe	0.043	97.79	35.28
		Peak2-China	8.76	45.29	37.68
	NL	Peak1	1.13	38.68	20.93
		Peak2	2.22	109.30	54.27
	SL	Peak1	24.19	28.97	12.79
		Peak2	4.28	51.17	42.24
	SM	Peak1	1.45	28.97	12.79
		Peak2	0.26	51.17	42.24
Grassland	NH	Peak1	17.15	16.2	10.94
		Peak2	5.87	29.58	24.17
	NM	Peak1	24.57	15.18	9.17
		Peak1-Europe	0.73	28.54	11.64

		Peak1-China	29.24	15.62	9.16
		Peak2	3.89	24.2	16.62
		Peak2-Europe	0.32	44.62	17.39
		Peak2-China	4.04	23.79	15.24
	NL	Peak1	16.77	21.99	18.22
		Peak2	3.49	53.23	15.98
	SL	Peak1	17.15	16.2	10.94
		Peak2	5.87	29.58	24.17
	SM	Peak1	6.62	16.2	10.94
		Peak2	2.56	29.58	24.17
Desert	World	Peak1	3.64	8.79	3.97
		Peak2	2.38	14.81	7.59
Cropland	NH	Peak1	119.90	21.94	15.51
		Peak2	19.99	59.69	53.35
	NM	Peak1	150.03	23.48	16.13
		Peak2	19.94	61.63	60.26
	NL	Peak1	91.91	19.75	13.78
		Peak2	18.87	57.95	53.61
	SL	Peak1	119.90	21.94	15.51
		Peak2	19.99	59.69	53.35
	SM	Peak1	119.90	21.94	15.51

	Peak2	19.99	59.69	53.35

Table S7 Parameterized HONO source mechanisms included in the model.

Sources	HONO formation reactions	Formula	Parameter range	Parameters in this study
Vehicle emissions [14]	The ratio of HONO to NOx from traffic sources	2.3% NO_x of transportation sources	0.29% –2.3% [20,54-56]	2.3%
Biomass burning	The ratio of HONO to NOx from biomass burning	0.23 NO_x of t biomass burning	0.025–0.23 [57-59]	0.23
Homogeneous reaction [14]	NO + OH→ HONO	CAM-Chem default		
NO2 heterogeneous reaction	NO_2 + aerosol + RH → HONO	$k = \frac{1}{4} \times v_{NO_2} \times Sa \times \gamma_{NO_2} \times f_{RH}$ *	γ_{NO_2} in the range of 1×10^{-6}– 2.6×10^{-5} [21,60,61]	8×10^{-6}
	NO_2 + aerosol + RH+ hv→ HONO	$k = \frac{1}{4} \times v_{NO_2} \times Sa \times \gamma_{NO_2} \times f_{RH} \times \frac{light\ intensity}{400}$ **	γ_{NO_2} in the range of 1.35×10^{-5}– 1.3×10^{-4} [60,62]	1×10^{-4}
	NO_2 + ground + RH → HONO	$k = \frac{1}{8} \times v_{NO_2} \times \frac{2 \times LAI}{H} \times \gamma_{NO_2} \times f_{RH}$	γ_{NO_2} in the range of 1×10^{-6}– 1×10^{-5} [19,63,64]	8×10^{-6}
	NO_2 + ground + RH+ hv→ HONO	$k = \frac{1}{8} \times v_{NO_2} \times \frac{2 \times LAI}{H} \times \gamma_{NO_2} \times f_{RH} \times \frac{light\ intensity}{400}$ **	γ_{NO_2} in the range of 2×10^{-6}– 6×10^{-5} [65-67]	6×10^{-5}
Particulate NO_3^- photolysis [14,68]	NO_3^- + hv→ HONO	$J_{pNO_3} = EF \times J_{HNO_3(CAM-Chem)}$	1–120 [14,68,69]	$\frac{8.3 \times 10^{-5}}{7 \times 10^{-7}}$

$$* \ f_{RH} = \begin{cases} \frac{RH}{50} & (RH < 50) \\ \frac{RH}{10} - 4 & (50 \le RH \le 80) \\ 4 & (RH \ge 80) \end{cases}$$

** To account for the photo enhancing effect, we set 1×10^{-4} for γ_{NO_2} during daytime conditions when the light intensity was below 400 W m^{-2}; when the light intensity exceeded 400 W m^{-2}, we adjusted it linearly in proportion to solar radiation.

Table S8 Differences in the impact of soil HONO emissions on air quality with different parameter selections (sensitivity study). The data listed in the table represent the increase in pollutant concentrations (%) due to soil HONO emissions.

	Species	Global	North America	South America	Europe	Africa	India	China	Australia
Parameters in this study	HONO	302.7	453.8	324.0	331.7	287.8	800.0	106.5	1243.5
	O_3	2.5	1.8	8.3	2.2	9.0	5.1	2.9	14.5
	OH	7.3	12.4	18.3	14.8	27.1	19.5	8.0	51.9
maximum value of the parameters	HONO	263.9	397.8	306.8	262.6	276.9	652.5	79.0	1213.0
	O_3	2.5	1.8	8.3	2.2	9.0	5.1	2.9	14.5
	OH	7.3	12.4	18.3	14.8	27.1	19.6	8.0	51.9
Minimum value of the parameter	HONO	1569.1	1986.5	2084.5	1538.2	2115.2	4252.9	482.5	6983.8
	O_3	2.7	2.4	8.8	2.8	9.6	5.9	2.6	18.4
	OH	8.0	14.9	19.7	16.9	29.7	22.9	7.4	58.1

Supplementary References:

1 Wang, J., Zhang, X., Guo, J., Wang, Z. & Zhang, M. Observation of nitrous acid (HONO) in Beijing, China: Seasonal variation, nocturnal formation and daytime budget. *Sci Total Environ* **587-588**, 350-359, doi:10.1016/j.scitotenv.2017.02.159 (2017).

2 Li, D. *et al.* Characteristics and sources of nitrous acid in an urban atmosphere of northern China: Results from 1-yr continuous observations. *Atmospheric Environment* **182**, 296-306, doi:10.1016/j.atmosenv.2018.03.033 (2018).

3 IGBP-DIS SoilData (V.0). A program for creating global soil-property databases. *IGBP Global Soils Data Task, France* (1998).

4 Stutz, J. *et al.* UV-visible absorption cross sections of nitrous acid. *Journal of Geophysical Research: Atmospheres* **105**, 14585-14592,

doi:10.1029/2000jd900003 (2000).
5 Li, X. *et al.* Revisiting the Ultraviolet Absorption Cross Section of Gaseous Nitrous Acid (HONO): New Insights for Atmospheric HONO Budget. *Environ Sci Technol* **58**, 4247-4256, doi:10.1021/acs.est.3c08339 (2024).
6 Li, X. *et al.* Exploring the atmospheric chemistry of nitrous acid (HONO) at a rural site in Southern China. *Atmospheric Chemistry and Physics* **12**, 1497-1513, doi:10.5194/acp-12-1497-2012 (2012).
7 Yang, Y. *et al.* Elucidating the effect of HONO on O3 pollution by a case study in southwest China. *Sci Total Environ* **756**, 144127, doi:10.1016/j.scitotenv.2020.144127 (2021).
8 Gu, R. *et al.* Atmospheric nitrous acid (HONO) at a rural coastal site in North China: Seasonal variations and effects of biomass burning. *Atmospheric Environment* **229**, doi:10.1016/j.atmosenv.2020.117429 (2020).
9 Yang, J. *et al.* Strong marine-derived nitrous acid (HONO) production observed in the coastal atmosphere of northern China. *Atmospheric Environment* **244**, doi:10.1016/j.atmosenv.2020.117948 (2021).
10 Sun, M., Cui, J. n., Zhao, X. & Zhang, J. Impacts of precursors on peroxyacetyl nitrate (PAN) and relative formation of PAN to ozone in a southwestern megacity of China. *Atmospheric Environment* **231**, doi:10.1016/j.atmosenv.2020.117542 (2020).
11 Liu, Y. *et al.* Semi-quantitative understanding of source contribution to nitrous acid (HONO) based on 1 year of continuous observation at the SORPES station in eastern China. *Atmospheric Chemistry and Physics* **19**, 13289-13308, doi:10.5194/acp-19-13289-2019 (2019).
12 Bernard, F. *et al.* Measurements of nitrous acid (HONO) in urban area of Shanghai, China. *Environ Sci Pollut Res Int* **23**, 5818-5829, doi:10.1007/s11356-015-5797-4 (2016).
13 Cheng, P. *et al.* An online monitoring system for atmospheric nitrous acid (HONO) based on stripping coil and ion chromatography. *J Environ Sci (China)* **25**, 895-907, doi:10.1016/s1001-0742(12)60251-4 (2013).
14 Fu, X., Wang, T., Zhang, L., Li, Q. & Wang, Z. The significant contribution of HONO to secondary pollutants during a severe winter pollution event in southern China. *Atmospheric Chemistry Physics* **19**, 1-14 (2019).
15 Hou, S., Tong, S., Ge, M. & An, J. Comparison of atmospheric nitrous acid during severe haze and clean periods in Beijing, China. *Atmospheric Environment* **124**, 199-206, doi:10.1016/j.atmosenv.2015.06.023 (2016).
16 Xu, W. *et al.* NH3-promoted hydrolysis of NO2 induces explosive growth in HONO. *Atmospheric Chemistry and Physics* **19**, 10557-10570, doi:10.5194/acp-19-10557-2019 (2019).
17 Ma, X. *et al.* Winter photochemistry in Beijing: Observation and model simulation of OH and HO(2) radicals at an urban site. *Sci Total Environ* **685**, 85-95, doi:10.1016/j.scitotenv.2019.05.329 (2019).
18 Nan, J., Wang, S., Guo, Y., Xiang, Y. & Zhou, B. Study on the daytime OH radical and implication for its relationship with fine particles over megacity of

Shanghai, China. *Atmospheric Environment* **154**, 167-178, doi:10.1016/j.atmosenv.2017.01.046 (2017).
19 Shi, X. *et al.* Budget of nitrous acid and its impacts on atmospheric oxidative capacity at an urban site in the central Yangtze River Delta region of China. *Atmospheric Environment* **238**, doi:10.1016/j.atmosenv.2020.117725 (2020).
20 Xu, Z. *et al.* Nitrous acid (HONO) in a polluted subtropical atmosphere: Seasonal variability, direct vehicle emissions and heterogeneous production at ground surface. *Atmospheric Environment* **106**, 100-109, doi:10.1016/j.atmosenv.2015.01.061 (2015).
21 Zheng, J. *et al.* Contribution of nitrous acid to the atmospheric oxidation capacity in an industrial zone in the Yangtze River Delta region of China. *Atmospheric Chemistry and Physics* **20**, 5457-5475, doi:10.5194/acp-20-5457-2020 (2020).
22 Meusel, H. *et al.* Daytime formation of nitrous acid at a coastal remote site in Cyprus indicating a common ground source of atmospheric HONO and NO. *Atmospheric Chemistry and Physics* **16**, 14475-14493, doi:10.5194/acp-16-14475-2016 (2016).
23 Lee, J. D. *et al.* Detailed budget analysis of HONO in central London reveals a missing daytime source. *Atmospheric Chemistry and Physics* **16**, 2747-2764, doi:10.5194/acp-16-2747-2016 (2016).
24 Michoud, V. *et al.* Study of the unknown HONO daytime source at a European suburban site during the MEGAPOLI summer and winter field campaigns. *Atmospheric Chemistry and Physics* **14**, 2805-2822, doi:10.5194/acp-14-2805-2014 (2014).
25 VandenBoer, T. C. *et al.* Evidence for a nitrous acid (HONO) reservoir at the ground surface in Bakersfield, CA, during CalNex 2010. *Journal of Geophysical Research: Atmospheres* **119**, 9093-9106, doi:10.1002/2013jd020971 (2014).
26 Kim, S. *et al.* Impact of isoprene and HONO chemistry on ozone and OVOC formation in a semirural South Korean forest. *Atmospheric Chemistry and Physics* **15**, 4357-4371, doi:10.5194/acp-15-4357-2015 (2015).
27 Ren, X. *et al.* A relaxed eddy accumulation system for measuring vertical fluxes of nitrous acid. *Atmospheric Measurement Techniques* **4**, 2093-2103, doi:10.5194/amt-4-2093-2011 (2011).
28 Nakashima, Y., Sadanaga, Y., Saito, S., Hoshi, J. & Ueno, H. Contributions of vehicular emissions and secondary formation to nitrous acid concentrations in ambient urban air in Tokyo in the winter. *Sci Total Environ* **592**, 178-186, doi:10.1016/j.scitotenv.2017.03.122 (2017).
29 Yu, Y. *et al.* Observations of high rates of NO2-HONO conversion in the nocturnal atmospheric boundary layer in Kathmandu, Nepal. *Atmospheric Chemistry and Physics* **9**, 6401–6415 (2009).
30 Wong, K. W., Oh, H. J., Lefer, B. L., Rappenglück, B. & Stutz, J. Vertical profiles of nitrous acid in the nocturnal urban atmosphere of Houston, TX. *Atmospheric Chemistry and Physics* **11**, 3595-3609, doi:10.5194/acp-11-3595-

2011 (2011).
31 Kleffmann, J. Daytime formation of nitrous acid: A major source of OH radicals in a forest. *Geophysical Research Letters* **32**, doi:10.1029/2005gl022524 (2005).
32 Kleffmann, J. *et al.* Measured and simulated vertical profiles of nitrous acid—Part I: Field measurements. *Atmospheric Environment* **37**, 2949-2955 (2003).
33 Acker, K., Möller, D., Wieprecht, W., Meixner, F. X. & Berresheim, H. Strong daytime production of OH from HNO2 at a rural mountain site. *Geophysical Research Letters* **33** (2006).
34 Elshorbany, Y. F. *et al.* Oxidation capacity of the city air of Santiago, Chile. *Atmos. Chem. Phys.,* **9**, 2257-2273 (2009).
35 Michoud, V. *et al.* Radical budget analysis in a suburban European site during the MEGAPOLI summer field campaign. *Atmospheric Chemistry and Physics* **12**, 11951-11974, doi:10.5194/acp-12-11951-2012 (2012).
36 Villena, G. *et al.* Nitrous acid (HONO) during polar spring in Barrow, Alaska: A net source of OH radicals? *Journal of Geophysical Research* **116**, doi:10.1029/2011jd016643 (2011).
37 Wentzell, J. J. B., Schiller, C. L. & Harris, G. W. Measurements of HONO during BAQS-Met. *Atmospheric Chemistry and Physics* **10**, 12285-12293, doi:10.5194/acp-10-12285-2010 (2010).
38 Zhou, X., Huang, G., Civerolo, K., Roychowdhury, U. & Demerjian, K. L. Summertime observations of HONO, HCHO, and O3at the summit of Whiteface Mountain, New York. *Journal of Geophysical Research* **112**, doi:10.1029/2006jd007256 (2007).
39 Oswald, R. *et al.* HONO emissions from soil bacteria as a major source of atmospheric reactive nitrogen. *Science* **341**, 3 (2013).
40 Wu, D. *et al.* Soil HONO emissions at high moisture content are driven by microbial nitrate reduction to nitrite: tackling the HONO puzzle. *The ISME Journal* **13**, 1688-1699, doi:10.1038/s41396-019-0379-y (2019).
41 Wu, D. *et al.* Global and regional patterns of soil nitrous acid emissions and their acceleration of rural photochemical reactions. *Journal of Geophysical Research: Atmospheres* **127**, e2021JD036379, doi:10.1029/2021jd036379 (2022).
42 Maljanen, M., Yli-Pirilä, P., Hytönen, J., Joutsensaari, J. & Martikainen, P. J. Acidic northern soils as sources of atmospheric nitrous acid (HONO). *Soil Biology and Biochemistry* **67**, 94-97, doi:10.1016/j.soilbio.2013.08.013 (2013).
43 Meusel, H. *et al.* Emission of nitrous acid from soil and biological soil crusts represents an important source of HONO in the remote atmosphere in Cyprus. *Atmospheric Chemistry and Physics* **18**, 799-813, doi:10.5194/acp-18-799-2018 (2018).
44 Mushinski, R. M. *et al.* Microbial mechanisms and ecosystem flux estimation for aerobic NOy emissions from deciduous forest soils. *Proceedings of the National Academy of Sciences of the United States of America* **116**, 2138-2145, doi:10.1073/pnas.1814632116 (2019).
45 Bao, F. *et al.* Key Role of Equilibrium HONO Concentration over Soil in

Quantifying Soil-Atmosphere HONO Fluxes. *Environ Sci Technol* **56**, 2204-2212, doi:10.1021/acs.est.1c06716 (2022).

46 Scharko, N. K. *et al.* Combined flux chamber and genomics approach links nitrous acid emissions to ammonia oxidizing bacteria and archaea in urban and agricultural soil. *Environmental Science Technology* **49**, 13825-13834 (2015).

47 Bhattarai, H. R., Virkajärvi, P., Yli-Pirilä, P. & Maljanen, M. Emissions of atmospherically important nitrous acid (HONO) gas from northern grassland soil increases in the presence of nitrite (NO_2^-). *Agriculture, Ecosystems & Environment* **256**, 194-199, doi:10.1016/j.agee.2018.01.017 (2018).

48 Sörgel, M., Trebs, I., Wu, D. & Held, A. A comparison of measured HONO uptake and release with calculated source strengths in a heterogeneous forest environment. *Atmospheric Chemistry and Physics* **15**, 9237-9251, doi:10.5194/acp-15-9237-2015 (2015).

49 Tang, K. *et al.* An automated dynamic chamber system for exchange flux measurement of reactive nitrogen oxides (HONO and NOX) in farmland ecosystems of the Huaihe River Basin, China. *Sci Total Environ* **745**, 140867, doi:10.1016/j.scitotenv.2020.140867 (2020).

50 Tang, K. *et al.* A dual dynamic chamber system based on IBBCEAS for measuring fluxes of nitrous acid in agricultural fields in the North China Plain. *Atmospheric Environment* **196**, 10-19, doi:10.1016/j.atmosenv.2018.09.059 (2019).

51 Xue, C. *et al.* Development and application of a twin open-top chambers method to measure soil HONO emission in the North China Plain. *Science of the Total Environment* **659**, 621-631, doi:10.1016/j.scitotenv.2018.12.245 (2019).

52 Wang, Y. *et al.* Large contribution of nitrous acid to soil-emitted reactive oxidized nitrogen and its effect on air quality. *Environ Sci Technol* **57**, 3516-3526, doi:10.1021/acs.est.2c07793 (2023).

53 Wang, Y. *et al.* Agricultural fertilization aggravates air pollution by stimulating soil nitrous acid emissions at high soil moisture. *Environ Sci Technol* **55**, 14556-14566, doi:10.1021/acs.est.1c04134 (2021).

54 Gutzwiller, L., F. Arens, Baltensperger, U., Gäggeler, H. W. & Ammann, M. Significance of semivolatile diesel exhaust organics for secondary HONO formation. *Environ. Sci. Technol.* **36**, 677–682, doi:doi:10.1021/es015673b (2002).

55 Kurtenbach, R. *et al.* Investigations of emissions and heterogeneous formation of HONO in a road traffic tunnel. *Atmospheric Environment* **35**, 3385-3394 (2001).

56 Kirchstetter, T. W., Harley, R. A. & Littlejohn, D. Measurement of nitrous acid in motor vehicle exhaust. *Environ. Sci. Technol.* **30**, 2843–2849, doi:doi:10.1021/es960135y (1996).

57 Burling, I. R. *et al.* Laboratory measurements of trace gas emissions from biomass burning of fuel types from the southeastern and southwestern United States. *Atmospheric Chemistry and Physics* **10**, 11115-11130, doi:10.5194/acp-10-11115-2010 (2010).

58 Akagi, S. K. *et al.* Emission factors for open and domestic biomass burning for use in atmospheric models. *Atmospheric Chemistry and Physics* **11**, 4039-4072, doi:10.5194/acp-11-4039-2011 (2011).
59 Keene, W. C. *et al.* Emissions of major gaseous and particulate species during experimental burns of southern African biomass. *Journal of Geophysical Research* **111**, doi:10.1029/2005jd006319 (2006).
60 Xue, C. *et al.* HONO Budget and Its Role in Nitrate Formation in the Rural North China Plain. *Environ Sci Technol* **54**, 11048-11057, doi:10.1021/acs.est.0c01832 (2020).
61 Zhang, X. *et al.* Elucidating HONO formation mechanism and its essential contribution to OH during haze events. *npj Climate and Atmospheric Science* **6**, doi:10.1038/s41612-023-00371-w (2023).
62 Xue, C. *et al.* Atmospheric measurements at Mt. Tai – Part II: HONO budget and radical (RO_{<i>x</i>} + NO<sub>3</sub >) chemistry in the lower boundary layer. *Atmospheric Chemistry and Physics* **22**, 1035-1057, doi:10.5194/acp-22-1035-2022 (2022).
63 Gu, R. *et al.* Nitrous acid in the polluted coastal atmosphere of the South China Sea: Ship emissions, budgets, and impacts. *Sci Total Environ* **826**, 153692, doi:10.1016/j.scitotenv.2022.153692 (2022).
64 Zhang, W. *et al.* Aging of pollution air parcels acts as the dominant source for nocturnal HONO. *Sci Total Environ* **881**, 163438, doi:10.1016/j.scitotenv.2023.163438 (2023).
65 Zhang, X. *et al.* The Levels and Sources of Nitrous Acid (HONO) in Winter of Beijing and Sanmenxia. *Journal of Geophysical Research: Atmospheres* **127**, doi:10.1029/2021jd036278 (2022).
66 Liu, J. *et al.* Detailed budget analysis of HONO in Beijing, China: Implication on atmosphere oxidation capacity in polluted megacity. *Atmospheric Environment* **244**, doi:10.1016/j.atmosenv.2020.117957 (2021).
67 Liu, Y. *et al.* A comprehensive model test of the HONO sources constrained to field measurements at rural North China Plain. *Environmental Science & Technology* **53**, 3517-3525, doi:10.1021/acs.est.8b06367 (2019).
68 Ye, C., Zhang, N., Gao, H. & Zhou, X. Photolysis of particulate nitrate as a source of HONO and NOx. *Environmental Science & Technology* **51**, 6849-6856, doi:10.1021/acs.est.7b00387 (2017).
69 Romer, P. S. *et al.* Constraints on Aerosol Nitrate Photolysis as a Potential Source of HONO and NOx. *Environ Sci Technol* **52**, 13738-13746, doi:10.1021/acs.est.8b03861 (2018).
